# Validity and Reliability of the Upper Extremity Work Demands Scale

**DOI:** 10.1007/s10926-016-9683-9

**Published:** 2016-11-15

**Authors:** Nora W. Jacobs, Redmar J. Berduszek, Pieter U. Dijkstra, Corry K. van der Sluis

**Affiliations:** 1 Department of Rehabilitation Medicine, University Medical Center Groningen, University of Groningen, Groningen, The Netherlands; 2 Department of Oral and Maxillofacial Surgery, University Medical Center Groningen, University of Groningen, Groningen, The Netherlands

**Keywords:** Work, Occupational exposure, Upper extremity, Self report, Reproducibility of results

## Abstract

*Purpose* To evaluate validity and reliability of the upper extremity work demands (UEWD) scale. *Methods* Participants from different levels of physical work demands, based on the Dictionary of Occupational Titles categories, were included. A historical database of 74 workers was added for factor analysis. Criterion validity was evaluated by comparing observed and self-reported UEWD scores. To assess structural validity, a factor analysis was executed. For reliability, the difference between two self-reported UEWD scores, the smallest detectable change (SDC), test–retest reliability and internal consistency were determined. *Results* Fifty-four participants were observed at work and 51 of them filled in the UEWD twice with a mean interval of 16.6 days (SD 3.3, range = 10–25 days). Criterion validity of the UEWD scale was moderate (r = .44, *p* = .001). Factor analysis revealed that ‘force and posture’ and ‘repetition’ subscales could be distinguished with Cronbach’s alpha of .79 and .84, respectively. Reliability was good; there was no significant difference between repeated measurements. An SDC of 5.0 was found. Test–retest reliability was good (intraclass correlation coefficient for agreement = .84) and all item-total correlations were >.30. There were two pairs of highly related items. *Conclusion* Reliability of the UEWD scale was good, but criterion validity was moderate. Based on current results, a modified UEWD scale (2 items removed, 1 item reworded, divided into 2 subscales) was proposed. Since observation appeared to be an inappropriate gold standard, we advise to investigate other types of validity, such as construct validity, in further research.

## Introduction

Physical work demands are associated with the development of upper extremity musculoskeletal disorders [1–3]. Since the prevalence of complaints of arm, neck or shoulders (CANS) is substantial (36.8%) [4], it seems useful to gain insight into workload of the upper limbs. Several methods exist to assess physical workload. The Dictionary of Occupational Titles (DOT) classification [5] can be used to estimate upper extremity work demands [6]. The DOT subdivides jobs into 5 groups: sedentary, light, medium, heavy and very heavy work. However, this classification appeared to be invalid for assessing upper extremity work demands [7]. Since the DOT subdivision is based on general physical work effort, it is imaginable that it less applicable to classify upper limb activities. Observation could provide a more accurate estimation of upper limb work demands, as it reflects actual work exposure. A few observational instruments have been developed to measure upper extremity work demands, e.g. Strain Index [8], Rapid Upper-Limb Assessment [9], and American conference of governmental industrial hygienists Threshold Limit Value for Hand Activity Level (ACGIH HAL-TLV) [10]. With the use of these instruments, observers acquire insight into clearly visible body postures and work activities [11]. However, those instruments often capture only a limited amount of possible exposures of the upper extremity. Besides, the need of an observer makes these instruments expensive, time-consuming and less suitable for application in clinical practice or in large epidemiological studies.

Self-reported work exposures might be a useful alternative to observational methods. Self-reports can provide a simple and cost-effective estimate of physical demands. Workers seem to be able to accurately report time spent in general work tasks performed with their upper extremities [12, 13]. Several questionnaires to assess work exposures exist, but often their measurement properties have not, or not properly, been tested [14, 15]. Surveys exclusively related to upper extremity use are limited [12]. To explore the validity of the DOT classification, a new questionnaire to measure upper extremity work demands was developed because of the lack of a suitable instrument [7]. All questions related to upper extremity work demand were selected from the Dutch musculoskeletal questionnaire (DMQ) to form the upper extremity work demands (UEWD) scale. The DMQ has been developed in 2001 to analyze general musculoskeletal workload and appears to have a fair convergent and divergent validity [16]. Measurement properties of the UEWD scale have not yet been evaluated. Therefore, the objectives of this study were to assess (1) validity and (2) reliability of the UEWD scale.

## Methods

### Participants

Participants of this cross-sectional study were recruited between September 2015 and November 2015. The DOT classification system was used to select workers from different levels of physical effort. The intention was to include employees from all 5 DOT categories in an equally distributed way. Participants were employees of the University Medical Center Groningen, the Ommelander Hospital Group Delfzijl and shipyard De Hoop in Foxhol, the Netherlands. Employees aged 18 years and over with sufficient understanding of the Dutch language to fill in the UEWD scale were included. Participants completed a short questionnaire regarding their education, work and health.

To be able to assess structural validity by using factor analysis, a historical database of UEWD data from 74 workers were added [17].

### Upper Extremity Work Demands (UEWD) Scale

The UEWD scale, as suggested by Opsteegh et al. [7], consisted of 7 items which should be rated on a 4-point Likert scale. The item ‘lift, push, pull or carry very heavy loads (>25 kg)’ was excluded since it correlated highly with the item ‘lift, push, pull or carry heavy loads (>5 kg)’. Opsteegh et al. did not include employees who were classified in DOT 5 (very heavy work). Since we aimed to include workers out of every DOT category, we decided to re-add the item, thereby creating an 8-item UEWD scale with total scores ranging from 8 (lowest upper extremity work demands) to 32 (highest upper extremity work demands) (Appendix, Table 4).

### Procedure

#### Validity

Criterion validity indicates the degree to which an instrument relates to a gold standard [18]. We used direct observation as gold standard: observed UEWD scores were compared with self-reported UEWD scores. For testing criterion validity at least 50 subjects should be included [19]. One researcher (NJ) visited all subjects at work to observe them for about 1 h while they performed their normal tasks. Real-time task analysis of all upper limb work activities was performed using PalmTRAC 2.5, a renewed version of the task recording and analysis on computer system which exists of a handheld device (Palm) and a PC application [20, 21]. The PC application was used to create a library consisting of multiple blocks of the 8 UEWD items and this library was transferred to the Palm. During observation, in each block a UEWD item could be selected, which made it possible to register simultaneously performed UEWD tasks. The selection of tasks on the Palm could be done in a fraction of a second. Every movement that conformed with a UEWD item was registered. Selection of item 1 and 2 (lift, push, pull or carry heavy (>5 kg) respectively very heavy (>25 kg) demands) was based on estimated weights; item 4 (bend/twist the wrists/hands) was selected when wrists were bended and item 5 (work in an awkward position with the wrists/hands during an extended period of time) was selected if work had to be done with bended wrists for a longer time; item 7 (keep your arms up) was selected if the hand was at or above shoulder level. For each UEWD item the observed exposure was calculated as percentage of the total observation time. The total observed exposure, calculated by summing the exposure percentages of the 8 items, ranged from 0% (no exposure to the items at all) to 100% (exposure to all items at the same time during the entire observation). After observation, subjects were asked whether they considered the observed work tasks as representative for their usual tasks.

Furthermore, an exploratory factor analysis was conducted to investigate the structural validity of the UEWD scale. For factor analysis it is suggested to include 7 subjects per item, with a minimum of 100 subjects [19]. Therefore 74 UEWD scores from a historical cohort [17] were added to the self-reported UEWD scores. The ‘lift, push, pull or carry very heavy loads (>25 kg)’ item was not taken into account in the factor analysis, since this item was not collected in the historical cohort [14].

#### Reliability

Reliability refers to the extent to which the measurement is free from measurement error and can be subdivided into three measurement properties: measurement error, test–retest reliability and internal consistency [18]. To explore the reliability of the UEWD scale, self-reported UEWD scores were collected twice with an interval of about two weeks. If necessary, a reminder to complete the second UEWD scale was sent two weeks after the first measurement. This interval of 2 weeks was considered to be short enough to ensure that work tasks would not have changed and long enough to prevent recall bias. To analyze measurement error and test–retest reliability, the minimum recommended sample size is 50 subjects [19]. Internal consistency was explored by assessing item-total and inter-item correlation of self-reported UEWD scores.

### Statistical Analyses

The statistical analyses were performed with IBM SPSS Statistics for Windows, version 22.0 (IBM Corp, Amonk, NY).

#### Validity

*Criterion Validity* To examine the relationship between the total score of the self-reported UEWD scale and the proportion of total observed exposure time, the Pearson correlation coefficient was calculated. If the correlation coefficient was at least .70, criterion validity was considered to be good [19].

*Structural Validity* A preliminary analysis was performed to ascertain that the data was suitable for factor analysis. The average of the communalities should be around .60 or higher [22] and the ratio of participants to items should be at least 10:1 [23]. Inter-item correlations were checked for too low (<.30) or too high (>.90) values. To avoid multicollinearity we ascertained that the determinant was >.00001. Sampling adequacy was tested with the Kaiser-Meyer-Olkin (KMO) measure (accepted if >.50) and Bartlett’s test of sphericity (considered sufficient if *p* < .05). Data were extracted with principal axis factoring method, since the aim was to describe underlying dimensions of the UEWD items [22, 23]. As recommended by Roberson et al., oblique rotation was preferred over orthogonal rotation if the correlation between the factors exceeded 10% [22]. To determine the number of extracted factors, the Kaiser criterion (eigenvalue > 1 rule) and the scree plot were used [22, 24]. It is assumed that Cronbach’s alpha is an adequate parameter to assess internal consistency [19]. The items of the subscales were considered to be sufficiently correlated if Cronbach’s alpha was between .70 and .95.

#### Reliability

A paired *t* test was performed to assess differences between the means of the first and second self-reported UEWD total scores. Measurement error: The standard error of the mean (SEM) was used to calculate the smallest detectable change (SDC): SDC = 1.96 × √2 × SEM [25]. The SDC should be smaller than the minimal important change (MIC) [19], however, no generally accepted MIC for the UEWD scale is available. Limits of agreement were presented using a Bland–Altman plot. Those limits are defined as the mean difference between repeated measurements ± 1.96 SD of the difference [26].

*Test*–*Retest Reliability* The intraclass correlation coefficient (ICC) for absolute agreement (two-way random effects model) was calculated, which takes into account differences between both subjects and time-points [25]. An ICC_agreement_ above .70 was considered to be satisfactory [19].

*Internal Consistency* Item-total correlations were evaluated to analyze the contribution of the items to the total score. An item with a Spearman’s correlation coefficient of less than .30 was considered to contribute too little [27]. To evaluate whether there were items in the UEWD scale that measured almost the same construct, inter-item correlation was calculated. If the Spearman’s correlation between two items was .70 or higher, it was assumed that one of them could be removed [27].

## Results

### Participant Characteristics

Observational and self-reported UEWD data from 54 employees were collected (Tables 1, 2). The mean time of observation was 52.5 min (SD 20.4). A second UEWD score was obtained from 51 participants, on average 16.6 days (SD 3.3, range = 10–25 days) after the first one. For the factor analysis, data of 128 participants were used (Table 1).

**Table 1:** Participant characteristics (n = 128)

	Participants (n = 54)		Participants included for factor analysis (n = 74)^f^	
	n	(%)	n	(%)
Gender (male)	24	44	53	72
*Educational level* ^a^				
Low	8	15	5	7
Medium	24	44	18	24
High	22	41	50	68
*Complaints of*				
Arms (hand, wrist, elbow, shoulder)^b^	14	26	18	24
Neck^c^	16	30	15	20
Back^d^	13	24	18	24
Legs^e^	6	11	NA	NA
Reduced work capacity due to complaints of arms/neck/back/legs	9	17	NA	NA

	Mean	SD	Mean	SD
Age (years)	42.6	13.6	44.2	12.2
Duration of employment (years)	15.0	13.0	NA	NA
Average work time (h/week)	32.3	9.3	34.6	9.4

*NA* not available

^a^Low = no education/primary school, medium = secondary school/vocational school, high = college/university; 1 missing from Postema et al. (1%)

^b^1 missing (2%)

^c^2 missing (4%)

^d^4 missing (7%)

^e^3 missing (6%)

^f^Data were kindly provided by Postema et al. [17]

**Table 2:** Participants per DOT category (n = 54)

DOT category (n)	DOT 1 Sedentary work (11)	DOT 2 Light work (12)	DOT 3 Medium work (18)	DOT 4 Heavy work (13)
Profession (n)^a^	Secretary (5) Receptionist (3) Researcher (3)	Physician (3) Caterer helper (1) Sales clerk, food (2) Assembler, laundry (2) Medical-laboratory technician (4)	Physical therapist (5) Nurse (6) Cleaner (2) Groundskeeper (1) Janitor (2) Cook (2)	Distributor (6) Stock clerk (1) Ship fitter (6)

*DOT* dictionary of occupational titles

^a^Occupational titles as found in the DOT classification system are given

### Validity

#### Criterion Validity

**Fig. 1:**
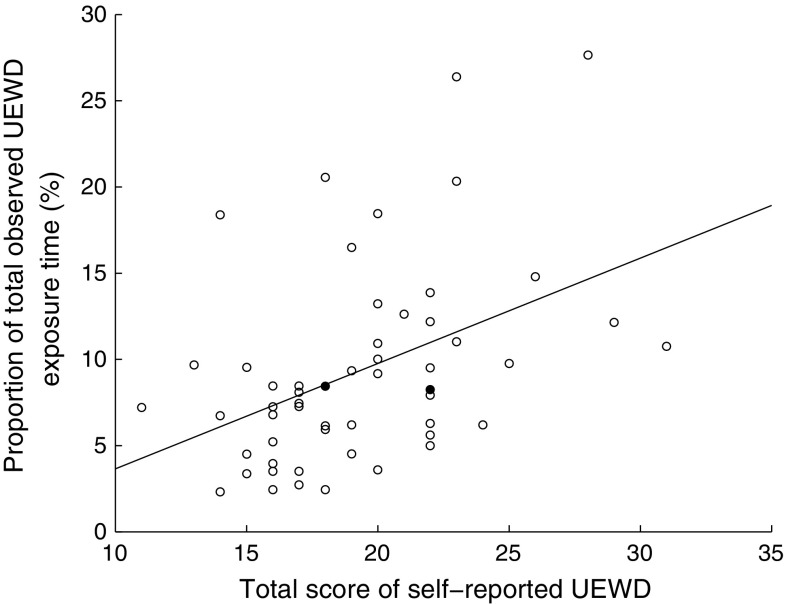
Total score of self-reported UEWD against proportion of total observed UEWD exposure time. *UEWD* upper extremity work demands, *open circle* representative observation (according to subject), *filled circle* unrepresentative observation (according to subject)

Almost all subjects (n = 52, 96%) considered the work they performed during the observation as representative for their usual work. The total score of the self-reported UEWD scale was significantly related to the total observed UEWD exposure, r = .44 (95% CI .2;.6, *p* = .001), explained variance 19% (Fig. 1). The correlation did not change after exclusion of both subjects with unrepresentative observations.

#### Structural Validity

**Fig. 2:**
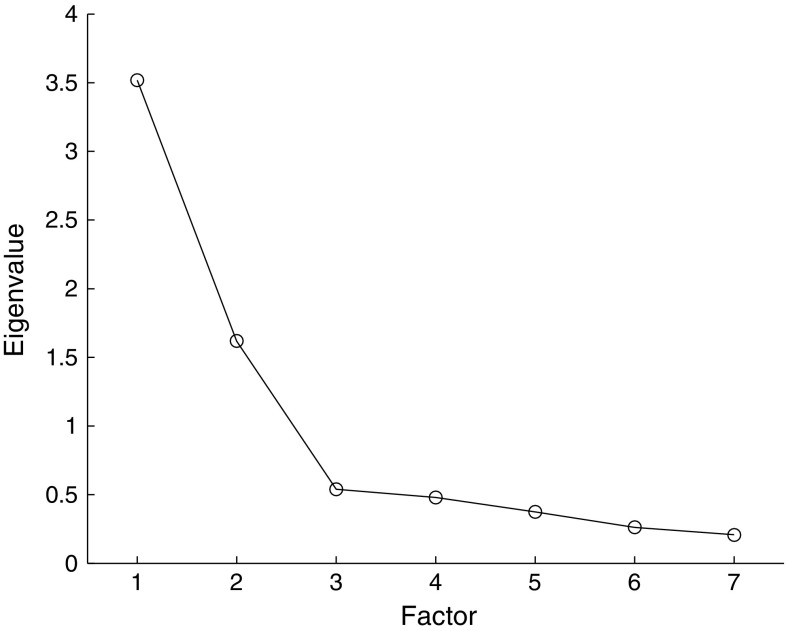
Scree plot of factor analysis of UEWD data

The average of the communalities was .64 and participant: item ratio was 128:7 = 18:1. All items had multiple correlations of at least .30 and that there were no correlations greater than .90. Multicollinearity was disproved since the determinant was .03. The overall KMO statistic was .79, the KMO values for individual items were .67 or higher. Bartlett’s test of sphericity was significant (*p* < .001). Both the Kaiser criterion and the scree plot justified the retaining of two factors (Fig. 2). Together, those factors explain 73.4% of the variance. Since the inter-factor correlation was .43, oblique (promax) rotation was used. Two factors can be distinguished: a ‘force and posture’ factor (Cronbach’s alpha .79) and a ‘repetition’ factor (Cronbach’s alpha .84) (Table 3).

**Table 3:** Summary of factor analysis results for the UEWD scale (n = 128)

	Factor 1 Force and posture	Factor 2 Repetition
*Rotated factor loadings* ^a^		
UEWD 1: lift, push, pull or carry heavy demands (>5 kg)	**.86**	−.18
UEWD 3: exert great force on tools or equipment	**.78**	−.16
UEWD 7: keep your arms up	**.70**	.04
UEWD 5: work in an awkward position with the wrists/hands during an extended period of time	**.50**	.34
UEWD 6: perform short repetitive movements with wrists/hands	−.01	**.93**
UEWD 8: make continuously similar movements with arms, hands or fingers every minute	−.23	**.90**
UEWD 4: bend/twist the wrists/hands	**.49**	**.52**
Eigenvalue	3.52	1.62
% of Variance	50.3	23.1
Cronbach’s alpha	0.79^b^	0.84^c^

UEWD 2 was excluded, as it was not evaluated by Postema et al. [17], extraction method: principal axis factoring, rotation method: promax with Kaiser normalization

*UEWD* upper extremity work demands

^a^Factor loadings > .40 appear in bold

^b^Included items: UEWD 1, 3, 5 and 7

^c^Included items: UEWD 4, 6 and 8

### Reliability

Mean total scores of the first and second self-reported UEWD scales were 19.24 (SD 4.1) and 19.47 (SD 4.8) respectively. The difference between those means was .23 (SD 2.6, 95% CI −1.0;.5, *p* = .52).

#### Measurement Error

**Fig. 3:**
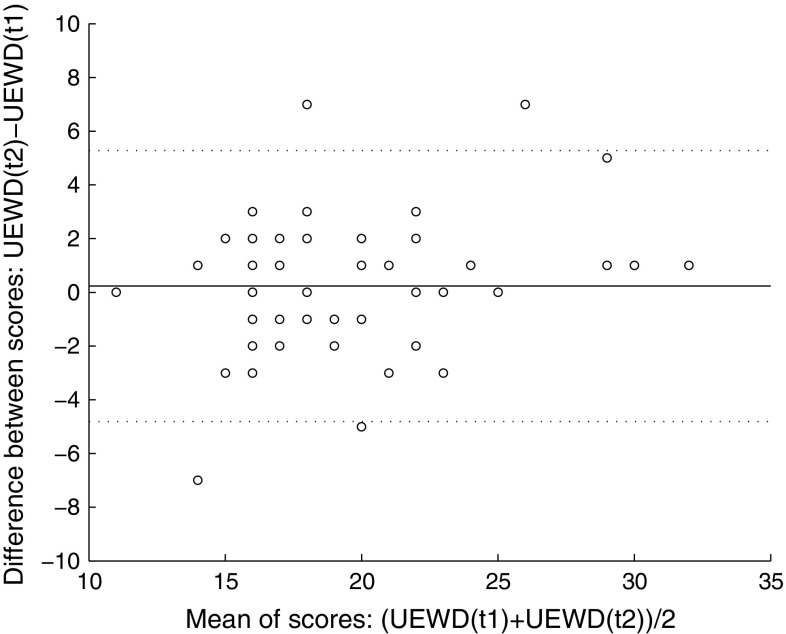
Bland-Altman plot of the difference between total scores of the first (t1) and second (t2) self-reported UEWD against the mean total scores of the first and second self-reported UEWD. *UEWD* upper extremity work demands

The SEM was 1.8 and the SDC 5.0. The limits of agreement (.23 ± 1.96 × 2.57 = 5.3 and -4.8) are presented in a Bland–Altman plot (Fig. 3). Ninety-three per cent of the difference points fell within those limits.

#### Test–Retest Reliability

The ICC_agreement_ was .8 (95% CI .7;.9).

#### Internal Consistency

The item-total correlations ranged from .38 to .79 (Appendix Table 5). The inter-item correlations between the ‘lift, push, pull or carry very heavy loads (>25 kg)’ item and the ‘lift, push, pull or carry heavy loads (>5 kg)’ item and between the ‘perform short repetitive movements with wrists or hands’ item and ‘make continuously similar movements with arms, hands or fingers every minute’ item were .71 and .81, respectively (Appendix Table 5).

## Discussion

A good reliability of the UEWD scale was found and factor analysis revealed that the scale can be subdivided into two subscales. However, criterion validity of the UEWD scale was moderate.

### Validity

#### Criterion Validity

The correlation between self-reported and observed UEWD scores (.44) was clearly below the recommended minimum correlation of .70 [19] and therefore indicates a moderate criterion validity [14]. Contrary to initial assumptions observation might not have been an appropriate gold standard. We experienced that it was hard to record small movements accurately, which was also noticed in a previous study [11]. Besides, self-reported workload (based on average work demands) and observed workload might not have corresponded enough. Particularly if an employee performs a variety of tasks, our one-hour observation might not have provided a good representation of upper limb work demands of the entire job. We found only one other study that compared observed and self-reported workload of the upper extremities and this study also showed correlations below the .70 threshold (median correlation of .46 in patients and .38 in controls) [13]. A further potential explanation for the lack of agreement between observed and self-reported UEWD scores might be the two high inter-item correlations of the UEWD scale. Highly related items make it possible that the researcher and the subject scored the same activity differently. For example, the observer doubted sometimes whether to record an activity as item 6 (perform short repetitive movements with wrists/hands), item 8 (make continuously similar movements with arms, hands or fingers every minute), or as both items at the same time. Moreover, a difference between perceived and actual work demands might have reduced the correlation between observed and self-reported UEWD scores. Multiple factors have been found that contribute to this difference: short duration of tasks, high variability of tasks within a job and tasks involving small, specific movements or postures [12, 15].

#### Structural Validity

The inter-item correlations were low but significant thereby they were suitable for factor analysis [27]. Factor analysis manifested that 2 factors could be distinguished within the UEWD scale: a ‘force and posture’ factor and a ‘repetition’ factor. Both subscales provide information about the kind of work exposure, which might be relevant for clinical practice. UEWD item 4 (bend/twist the wrists/hands) loaded similarly on both factors. It was added to the ‘repetition’ factor, since its loading was slightly higher and the content fitted better with this factor too. Both factors had a Cronbach’s alpha value within the .70–.95 range, which means that their internal consistency is good.

### Reliability

#### Measurement Error

The found SDC (5.0) means that a change of at least 5 points is needed to detect a true difference in UEWD scores. Since the MIC of the UEWD score is unknown, we were unable to determine whether the SDC of the UEWD scale was sufficiently low. Regarding the Bland and Altman plot, 93% of the difference points fell within the limits of agreement. This fits the assumption that about 95% falls within those limits and thereby indicates that the measurements are interchangeable [26, 27].

#### Test–Retest Reliability

Only one study concerning self-reported upper extremity work demands previously reported reliability parameters (ICC or weighted Kappa), ranging from .24 to .69 [28], thus all below the recommended threshold of .70. Direct comparison with the UEWD scores is however not readily possible, as the study investigated reliability per question, whereas we explored reliability of the total UEWD scores.

#### Internal Consistency

Item-total correlations revealed that all items contributed sufficiently to discriminate between employees. By exploring inter-item correlation, two pairs of highly related items were found. First, item 1 [lift, push, pull or carry heavy loads (>5 kg)] correlated highly with item 2 [lift, push, pull or carry very heavy loads (>25 kg)], which is in accordance with the finding of Opsteegh et al. [7]. To be classified in the DOT 5 category, a worker has to handle objects >45 kg occasionally, and/or >23 kg frequently, and/or >9 kg constantly [5]. We appeared to be unable to include workers from DOT 5, probably because Dutch law prescribes that workers are allowed to carry maximally 23 kg [29], although the Dutch Center for Occupational Diseases (NCvB) states that 17% of the Dutch employees regularly have to lift more than 25 kg [30]. However, we suggest to remove item 2 from the UEWD scale because of the high correlation with item 1. By keeping item 1, all heavy loads of 5 kg and above will be registered, which also includes the very heavy loads (>25 kg) from item 2. The other highly correlated items were 6 (perform short repetitive movements with wrists/hands) and 8 (make continuously similar movements with arms, hands or fingers every minute). We contacted the developer of the DMQ, dr. Hildebrandt (TNO, the Netherlands), to verify the difference between those items. Item 6 belongs to a question that evaluates work load per body part, whereas item 8 was established to obtain an overall impression. Dr. Hildebrandt deemed it not necessary to keep both items in our UEWD scale selection and advised to retain only item 8, which covers the whole upper extremity.

### Strengths and Weaknesses

The primary strength of our study was the heterogeneity of our study population: we included both men and women from different ages, with and without complaints of the upper limbs and from all levels of work demands that are allowed in the Netherlands. This suggests that the results can be generalized to other situations. Furthermore, all observations were performed by the same researcher, which excludes variance due to differences between observers. Lastly, we used corresponding constructs to assess criterion validity.

There were some limitations of this study. First, we have doubts if our observation of work demands was a true gold standard. In comparison to video recordings, direct observation does not allow to assess the accuracy of the recordings. Concurrent video recording could have contributed to a more accurate registration, especially considering simultaneously performed tasks. Simultaneous recording of different types of exposures, such as posture and repetition, is difficult and might have led to underscoring. Furthermore, observations may have been missed, because the observer had to look at the computer during recording. To our knowledge, there is no method available to determine the actual work demands of the upper limbs. In the future, measurement of activities with body worn sensors can possibly be used as a gold standard [31]. Our observed UEWD score might have corresponded better with the self-reported UEWD score if we had used longer or multiple observations [15]. Also, the correlation might have been higher if we had asked the participants to fill in the UEWD scale for the tasks they performed while being observed, instead of for their general work tasks. Another limitation was the use of the DOT categories to select employees, since this system inadequately classifies upper extremity work demands [7]. Also, we did not succeed to include employees from the heaviest DOT category. Furthermore, forward and backward translation was not used in the original Dutch and English versions of the DMQ, from which the UEWD items were selected [7, 16]. A final limitation was that during the application of the UEWD, we received feedback from the participants that item 5 (work in an awkward position with the wrists/hands during an extended period of time) could be answered in multiple ways. Some subjects noticed that they wondered whether they had to report how often their wrists/hands were in an awkward position, or how often their body was in an awkward position while working with wrists/hands. Because of ambiguity of item 5, we suggest to change this item to ‘work with wrists/hands in an awkward position during an extended period of time’. In this way, the item more clearly involves the upper extremities (see Appendix Table 6 for the modified UEWD scale).

### Further Research

Moderate criterion validity was found in this study, which suggests that further research is necessary, for example using (a combination of) other methods or instruments as gold standard or a longer direct observation of a broader variety of work tasks combined with video recordings. Also, research on other types of validity, such as construct validity, is needed to be able to estimate upper extremity work demands. Such research could provide more information about the applicability of the UEWD scale.

To verify whether the UEWD data fit the two factor model, more data should be collected to perform a confirmatory factor analysis. Force exertion, awkward postures and repetition are all related to development of complaints of the upper limbs [1, 3]. For future research it is also interesting to investigate whether high UEWD scores are related to the presence or development of upper extremity musculoskeletal disorders.

## Conclusion

The UEWD scale provides reliable self-reported estimations of upper extremity work demands. The scale appeared to consist of 2 subscales with good internal consistency and can be reduced from 8 to 6 items because of inter-item correlations. Another item was reworded. A modified UEWD scale was presented. Criterion validity of the UEWD scale is moderate, but it seems currently unfeasible to prove satisfactory criterion validity of self-reported work exposure of the upper limbs as no true gold standard is available. Further research with a better selection of instruments reflecting the gold standard or research on other types of validity should determine whether the UEWD scale can be used to measure work demands of the upper extremity.

## Appendix

See Tables 4, 5 and 6.

**Table 4:** Version of upper extremity work demands scale that was used in this study

		Rarely or never	Sometimes	Often	Almost always
*During work, do you have to*					
1	Lift, push, pull or carry heavy demands (>5 kg)?	1	2	3	4
2	Lift, push, pull or carry very heavy demands (>25 kg)?	1	2	3	4
3	Exert great force on tools or equipment?	1	2	3	4
4	Bend/twist the wrists/hands?	1	2	3	4
5	Work in an awkward position with the wrists/hands during an extended period of time?	1	2	3	4
6	Perform short repetitive movements with wrists/hands?	1	2	3	4
7	Keep your arms up?	1	2	3	4
8	Make continuously similar movements with arms, hands or fingers every minute?	1	2	3	4

**Table 5:** Inter-item and item-total correlation of self-reported UEWD scale (n = 54)

	Item 1	Item 2	Item 3	Item 4	Item 5	Item 6	Item 7	Item 8	Total score
Item 1	1.00	.71*	.45**	.44**	.17	−.07	.41**	−.05	.64**
Item 2	.71**	1.00	.60**	.35**	.21	−.14	.47**	−.12	.60**
Item 3	.45**	.60**	1.00	.10	.28*	−.21	.20	−.20	.38**
Item 4	.44**	.35**	.10	1.00	.49**	.50**	.30**	.49**	.79**
Item 5	.17	.21	.28*	.49**	1.00	.36**	.37**	.26	.63**
Item 6	−.07	−.14	−.21	.50**	.36**	1.00	.07	.81**	.53**
Item 7	.41**	.47**	.20	.30*	.37**	.07	1.00	−.09	.53**
Item 8	−.05	−.12	−.20	.49**	.26	.81**	−.09	1.00	.48**
Total score	.64**	.60**	.38**	.79**	.63**	.53**	.53**	.48**	1.00

*UEWD* upper extremity work demands

* Correlation is significant at the .05 level (2-tailed)

** Correlation is significant at the .01 level (2-tailed)

**Table 6:** Modified Upper Extremity Work Demands scale, based on results from this study

		Rarely or never	Sometimes	Often	Almost always
During work, do you have to					
*Force and posture subscale*					
1	Lift, push, pull or carry heavy demands (>5 kg)?	1	2	3	4
2	Exert great force on tools or equipment?	1	2	3	4
3	Work with wrists/hands in an awkward position during an extended period of time?	1	2	3	4
4	Keep your arms up?	1	2	3	4
*Repetition subscale*					
5	Bend/twist the wrists/hands?	1	2	3	4
6	Make continuously similar movements with arms, hands or fingers every minute?	1	2	3	4

## Abbreviations

DMQ: Dutch musculoskeletal questionnaire
DOT: Dictionary of occupational titles
ICC: Intraclass correlation coefficient
KMO: Kaiser–Meyer–Olkin
MIC: Minimal important change
SDC: Smallest detectable change
SEM: Standard error of the mean
UEWD: Upper extremity work demands
